# Distribution and Transfer of Plasmid Replicon Families among Multidrug-Resistant *Enterococcus faecalis* and *Enterococcus faecium* from Poultry

**DOI:** 10.3390/microorganisms10061244

**Published:** 2022-06-17

**Authors:** Sohyun Cho, Elizabeth A. McMillan, John B. Barrett, Lari M. Hiott, Tiffanie A. Woodley, Sandra L. House, Jonathan G. Frye, Charlene R. Jackson

**Affiliations:** 1 Bacterial Epidemiology and Antimicrobial Resistance Research Unit, USDA-ARS, U.S. National Poultry Research Center, Athens, GA 30605, USA; sohyun.cho@usda.gov (S.C.); elizabeth.mcmillan@usda.gov (E.A.M.); bennybarrett09@gmail.com (J.B.B.); lari.hiott@usda.gov (L.M.H.); tiffanie.woodley@usda.gov (T.A.W.); sandra.house@usda.gov (S.L.H.); jonathan.frye@usda.gov (J.G.F.); 2 Oak Ridge Institute for Science and Education, Oak Ridge, TN 37830, USA

**Keywords:** *Enterococcus*, poultry, plasmids, conjugation, antimicrobial resistance

## Abstract

The presence and transfer of plasmids from commensal bacteria to more pathogenic bacteria may contribute to the dissemination of antimicrobial resistance. However, the prevalence of plasmids from commensal bacteria, such as the enterococci, in food animals remains largely unknown. In this study, the diversity and prevalence of plasmid families from multidrug-resistant (MDR; resistance to three or more antimicrobials) enterococci from poultry carcasses were determined. Plasmid-positive MDR enterococci were also tested for the ability to transfer plasmids to other enterococci using conjugation. MDR *Enterococcus faecalis* (*n* = 98) and *Enterococcus faecium* (*n* = 696) that were isolated from poultry carcass rinsates between 2004 and 2011 were tested for the presence of 21 plasmid replicon (*rep*) families using multiplex PCR. Approximately 48% of *E. faecalis* (47/98) and 16% of *E. faecium* (110/696) were positive for at least one *rep*-family. Fourteen *rep*-families were detected overall, and ten *rep*-families were shared between *E. faecalis* and *E. faecium*. The *rep*_7_ and *rep*_17_ families were unique to *E. faecalis*, while the *rep*_5_ and *rep*_8_ families were unique to *E. faecium*. The *rep*_9_ family was predominant in both *E. faecalis* and *E. faecium* for all the years tested. The greatest number of *rep*-families detected was in 2005 (*n* = 10), and the least was in 2009 (*n* = 1). Eight *rep*-families were transferred from *E. faecalis* donors to the *E. faecalis* JH2-2 recipient using conjugation. Results from this study showed that *E. faecalis* and *E. faecium* from poultry carcasses contain numerous and diverse *rep*-families that are capable of conjugal transfer.

## 1. Introduction

The *Enterococcus* spp. are Gram-positive bacteria that are commonly found in the intestinal flora of humans and animals [1,2,3]. They are also important opportunistic pathogens and are among the leading causes of healthcare-associated pathogens in the United States [4,5,6]. *Enterococcus faecalis* and *Enterococcus faecium* are the two clinically important species as they account for most enterococcal infections, which cause a variety of infections, including urinary tract infections, soft tissue infections, bacteremia, and endocarditis in humans [2]. Some factors that have made the enterococci a successful nosocomial pathogen are their intrinsic resistance to many antimicrobial agents and their ability to acquire resistance via horizontal gene transfer [2]. The widespread resistance among enterococci has also made them one of the sentinel organisms used to track antimicrobial resistance trends by the National Antimicrobial Resistance Monitoring System (NARMS), a partnership between the Centers for Disease Control and Prevention (CDC), the U.S. Food and Drug Administration (FDA), and the U.S. Department of Agriculture (USDA) [7]. From 1996 to 2011, the surveillance of bacterial isolates of animal origin was conducted by the USDA Agricultural Research Service (USDA-ARS), and from 2011 to the present, by the USDA Food Safety and Inspection Service (FSIS). Certain enteric bacteria are monitored by NARMS to track trends in antimicrobial resistance in humans, retail meats, and food animals in the United States, and the enterococci are included to monitor antimicrobial resistance trends in Gram-positive bacteria.

Antimicrobials administered to food animals for therapeutic or prophylactic purposes have resulted in the development of antimicrobial resistance in enterococci. While most enterococci are commensals and do not cause illnesses in food animals, enterococci commonly carry plasmids encoding antimicrobial resistance, and the transfer of such plasmids from enterococci to more pathogenic bacteria is cause for concern. Studies have shown that enterococci can disseminate their antimicrobial resistance genes to other bacteria, such as *Staphylococcus aureus*, through horizontal gene transfer [8,9]. Thus, enterococci in food animals serve as a reservoir of antimicrobial resistance, with the potential to transfer antimicrobial resistance to human pathogenic bacteria and spread to the human population through the consumption of animal products or through human–animal contact. Hence, it is important to study the prevalence of plasmids, especially those associated with antimicrobial resistance, in enterococci in food animals and their potential transfer to other bacteria.

In 2010, a system for the classification of plasmids for Gram-positive bacteria was determined by Jensen et al. [10]. The system was established by the comparison of sequences of replication-initiating genes (*rep*) as well as *rep*-like genes from Gram-positive plasmids available from GenBank; 21 replicon families (20 *rep*-families and a unique sequence) were determined. In this study, the distribution and prevalence of plasmid families from multidrug-resistant (MDR; resistance to three or more antimicrobials) *E. faecalis* and *E. faecium* from poultry, collected as part of NARMS, were determined using multiplex PCR developed from the analysis. Isolates representative of each plasmid prototype from detected *rep*-families were selected and used to determine if the plasmids could transfer to other enterococci using bacterial conjugation. Results from this study will provide information on *rep*-family prevalence, diversity, and mobility in MDR *E. faecalis* and *E. faecium* from poultry.

## 2. Materials and Methods

### 2.1. Bacterial Strains, Isolation, and Identification

The enterococci in this study were collected from healthy poultry carcass rinsates between 2004 and 2011 by the USDA-ARS as a part of the animal arm of NARMS, as previously described [11]. All enterococci were from poultry carcass rinsates collected from poultry processing facilities located in different geographical regions of the U.S. Excel was used to randomly select 100 multidrug-resistant (MDR; defined as resistance to three or more antimicrobials in this study) enterococci (either *E. faecalis* or *E. faecium*) from each study year. Briefly, 1 mL of rinsates were inoculated into 9 mL of BBL Enterococcosel broth (Becton Dickinson, Sparks, MD, USA) and incubated at 37 °C overnight. Positive cultures were then streaked onto BBL Enterococcosel agar and incubated at 37 °C overnight. A single colony of presumptive enterococcal isolates was sub-cultured onto slants of brain heart infusion agar (BHIA) (Becton Dickinson) for storage, followed by sub-culturing twice onto blood agar (trypticase soy agar containing 5% defibrinated sheep blood) for the isolation of pure colonies. Isolates were identified to species using multiplex PCR, as previously described [11].

### 2.2. Antimicrobial Susceptibility Testing

Minimum inhibitory concentrations (MIC; μg/mL) of enterococci were determined by broth microdilution according to the manufacturer’s directions using the Sensititre^TM^ semi-automated antimicrobial susceptibility system (Trek Diagnostic Systems, Inc., Cleveland, OH, USA) and the Sensititre^TM^ Gram-Positive Custom Plate CVM2AGPF or CMV3AGPF (chloramphenicol, ciprofloxacin, daptomycin, erythromycin, flavomycin, gentamicin, kanamycin, lincomycin, linezolid, nitrofurantoin, penicillin, streptomycin, Synercid (Quinupristin/Dalfopristin), tetracycline, tigecycline, tylosin, and vancomycin). Results were interpreted according to the Clinical and Laboratory Standards Institute (CLSI) guidelines, when defined [12,13], and NARMS (https://www.fda.gov/media/108180/download, accessed on 13 June 2022). No CLSI interpretive criteria were defined for kanamycin, lincomycin, and tylosin, and only susceptible breakpoints were established for tigecycline (≤0.25 μg/mL). *E. faecalis* ATCC 29212, *E. faecalis* ATCC 51299, *S. aureus* ATCC 29213, and *Escherichia coli* ATCC 25922 were used as quality controls for the determination of MIC.

### 2.3. Replicon Typing

Replicon families were determined, as previously described [10]. Six multiplex PCR and one additional PCR (targeting pUB101) were used to detect 21 defined Gram-positive replicon families. A template for the *rep*-family PCR was prepared by suspending a single bacterial colony in 100 μL of sterile deionized water; 5 μL of template was used in each amplification reaction.

### 2.4. Bacterial Matings

The transfer of plasmids from different replicon families was determined by filter mating, as previously described [14]. The donor strains were randomly selected based on the plasmid type present and were resistant to either tetracycline or erythromycin. The recipient strain used in the matings was *E. faecalis* JH2-2 (rifampicin, fusidic acid), and transconjugants were selected using media containing either tetracycline (16 μg/mL) or erythromycin (8 μg/mL) in combination with fusidic acid (25 μg/mL). *Rep*-family multiplex PCR was performed on transconjugants to confirm the presence of specific plasmid replicon types.

### 2.5. Statistical Analysis of Tetracycline Resistance and rep-Family

The ratio of tetracycline-susceptible and resistant enterococci that contain plasmid replicons, as determined by *rep*-family typing, was compared using 95% confidence intervals calculated usingExcel.

## 3. Results

### 3.1. Presence of rep-Families among E. faecalis and E. faecium

Of the 794 MDR enterococcal isolates selected for testing, 48% of *E. faecalis* (47/98) and 16% of *E. faecium* (110/696) were positive for at least one *rep*-family. Six *E. faecium* that were originally selected were excluded in the final analysis as the isolates were duplicates. Overall, the host range for the 21 *rep*-families tested was wide as 10 of the 21 were present in both *E. faecalis* and *E. faecium* (Table 1). Overall, 14 *rep*-families were detected, indicating the presence of 14 different plasmids among the isolates. Although 12 *rep*-families were found among *E. faecalis* and *E. faecium*, the host range was different. Two *rep*-families (*rep*_7_, plasmid prototype pUSA02 and *rep*_17_, plasmid prototype pRUM) were unique to *E. faecalis* isolates and not detected in *E. faecium*. Likewise, *rep*_5_ (pSAS/pN315) and *rep*_8_ (pAM373) were found in *E. faecium,* but not in *E. faecalis*. When examined by species, *E. faecalis* and *E. faecium* were each negative for nine *rep*-families; seven of those *rep*-families (*rep*_4_ (pMBB1), *rep*_10_ (pIM13), *rep*_10b_ (pSK6), *rep*_12_ (pBMB67), *rep*_13_ (pC194), *rep*_15_ (pUSA03), and *rep*_16_ (pSAS)) were not found in any of the isolates tested (Table 1).

### 3.2. Resistance Profiles among rep-Positive Enterococci

Antimicrobial resistance phenotypic patterns were largely variable among the isolates, with few dominant patterns observed for all the species and years examined (Table 2, Table 3, Table 4 and Table 5 and Table S1). The most common MDR pattern among the isolates was GenKanLinTet and LinNitTet (*n* = 5 for both), with GenKanLinTet only detected in *E. faecalis,* while LinNitTet was found in both *E. faecalis* and *E. faecium*. Due to the high number of different MDR patterns, no distinct pattern was associated with specific plasmid replicons. However, high numbers of plasmid-positive MDR *E. faecalis* (83%; 39/47) and *E. faecium* (76%; 84/110) were resistant to tetracycline. After 2005, isolates were not tested against bacitracin as this antibiotic was removed from the susceptibility plate due to widespread resistance in the enterococci. The lack of testing for bacitracin resistance in the subsequent years did not appear to affect the dominant MDR patterns.

### 3.3. Distribution of rep-Families over Time

**Figure 1:**
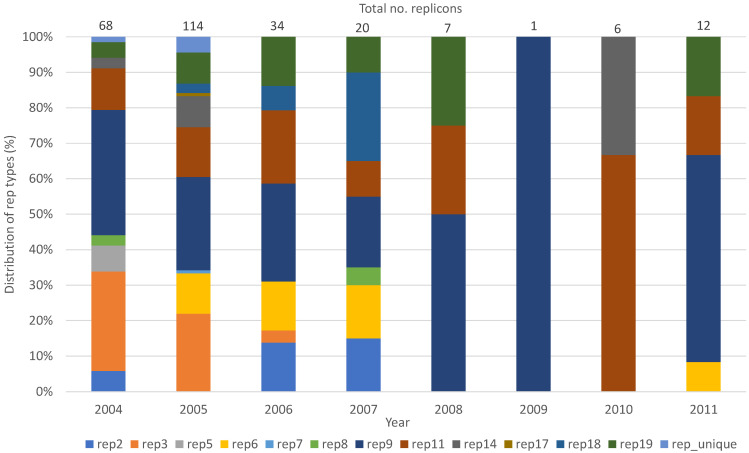
Distribution of *rep*-families detected in poultry enterococci (*E. faecalis* and *E. faecium*) from 2004 to 2011.

The *rep*_9_ family (*n* = 77), followed by *rep*_3_ (*n* = 45) and rep_11_ (*n* = 39), were the most prevalent *rep*-families in both *E. faecalis* and *E. faecium* for all the years tested (Table 2, Table 3, Table 4 and Table 5). Abundance of *rep*-families varied over the years, with the highest number of different *rep*-families detected in 2005 (*n* = 10) (Figure 1). The number of *rep*-families declined from the earlier years (2004–2007) to 2009, in which only one *rep*-family (*rep*_9_) was detected. The number of *rep*-families detected began to increase again in 2010 and 2011, in which two (*rep*_11_ and *rep*_14_) and four (*rep*_6_, *rep*_9_, *rep*_11_, *rep*_19_) different *rep*-families, respectively, were found (Figure 1).

### 3.4. Transfer of rep-Families

Twenty enterococci (10 each for *E. faecalis* and *E. faecium*) containing different plasmid replicons were selected as donors in conjugation studies. Tetracycline was used as the counter-selectable marker, except for one *E. faecalis* donor for which erythromycin was used. Conjugations using *E. faecium* as the donor resulted in either no transconjugants, suggesting that the transfer was unsuccessful, or in colonies that were negative for the tested plasmid replicons (data not shown). Eight *rep*-families (*rep*_3_, *rep*_6_, *rep*_7_, *rep*_9_, *rep*_17_, *rep*_18_, *rep*_19_, and *rep*_Unique_) were transferred from the *E. faecalis* donors to the *E. faecalis* JH2-2 recipient in the matings (Table 6). Two *E. faecalis* donors containing *rep*_1_ and *rep*_14_ families were characteristic of the *E. faecium* matings in that colonies were present, but no replicon transfer was detected (Table 6). No transfer of *rep*-family prototypes 1 (pIP501), 2 (pRE25), 8 (pAM373), 11 (pEF1071), 14 (pRI), or 16 (pSAS) was detected.

### 3.5. Frequency of rep-Families in Tetracycline-Resistant Enterococci

**Figure 2:**
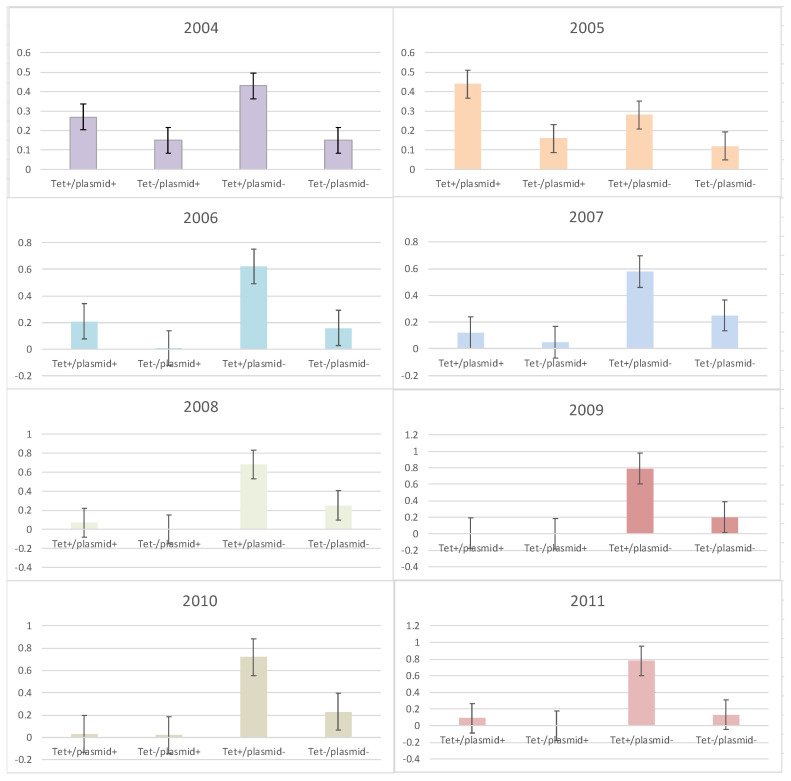
Frequency of tetracycline-susceptible and resistant enterococci with and without plasmid replicons from 2004 to 2011. Error bars reflect 95% confidence intervals.

The frequency of the presence or absence of *rep*-families in tetracycline-susceptible and resistant enterococci was determined for the years examined (Figure 2). It was only in 2005 when the frequency of tetracycline resistance and the presence of at least one plasmid replicon (tet+/plasmid+) were greater than tetracycline-resistant isolates without a plasmid (tet+/plasmid−) (Figure 2). For all other years, the opposite was observed as the frequency of tetracycline-resistant isolates without a plasmid replicon was higher than all other combinations (tet+/plasmid+, tet−/plasmid+ and tet−/plasmid−).

## 4. Discussion

Studies characterizing the antimicrobial resistance and plasmid content of bacterial strains of clinical origin are well-represented in the literature; however, knowledge of plasmid distribution for strains isolated from non-clinical sources such as food animals is largely unavailable. The use of antimicrobials in the food supply, including food animal production, coupled with the potential for transfer of antimicrobial-resistant bacteria into the human population, supports the need for additional data from non-clinical sources. As commensal bacteria such as enterococci contain plasmids that can harbor multiple resistances and are often mobilizable, the present study aimed to analyze the plasmid replicon content of *E. faecalis* and *E. faecium* collected from poultry during an eight-year period, in which the USDA-ARS participated as a part of NARMS. The analysis utilized a PCR-based plasmid replicon typing system designed to define plasmid replicon families from Gram-positive bacteria, including enterococci [10].

Of the replicon families examined in this study, ten of the *rep*-families were found in both *E. faecalis* and *E. faecium,* indicating a broad range of distribution. This observation was different from that previously described as only four *rep*-families were shared between *E. faecalis* and *E. faecium* based on information from PubMed and GenBank [10]. Limited information on the distribution of *rep*-families in enterococci can be attributed to the low number of strains examined, the limited sources of the strains (i.e., clinical sources), and specific strain characteristics such as certain antimicrobial resistance phenotypes or genotypes [15]. Although the present study specifically examined *E. faecalis* and *E. faecium* from poultry, a large collection was analyzed, and the different origins of the isolates allowed for a comparison to the clinical as well as non-clinical sources. Furthermore, biasing due to antimicrobial resistance to one specific antimicrobial was minimized by targeting MDR isolates, which allowed for a greater number of isolates to be included as well as a higher probability of isolates containing at least one plasmid replicon family.

About 20% of the total enterococcal isolates were positive for at least one *rep*-family. Previous studies on the plasmid classification of antimicrobial-resistant *Enterococcus* have shown that a considerable portion of *Enterococcus* from animal and environment sources (approximately 30%) did not harbor any plasmids from the 21 *rep*-families, while *Enterococcus* from human sources were mostly positive for *rep* genes [10,16,17,18,19,20]. This suggests that plasmids present in non-human enterococcal isolates are different from those found in enterococcal isolates of human origin and may be comprised of those not included in this classification system. It could also indicate that less antibiotic pressure in non-clinical settings may have resulted in the loss of plasmids.

It is interesting to note that the number of enterococcal isolates positive for *rep* genes decreased over time, as did the diversity of *rep*-families. About half of the isolates tested were identified to harbor plasmids in 2004 and 2005, while the number of the plasmid-positive isolates decreased to just one isolate in 2009. This was unexpected as the selective pressure of antibiotics would enhance the acquisition and exchange of resistance genes through various mechanisms, including horizontal gene transfer via plasmids. In the absence of antibiotics, plasmids encoding antimicrobial resistance tend to be lost as plasmid maintenance is a burden to the bacterial cell [21]. However, it is unlikely that selective conditions were absent in the poultry farms during the years the samples were taken, although changes in the class of antibiotics used cannot be ruled out. Antibiotics were still being used in food animals until their use in farm animals for growth promotion purposes was banned in the U.S. in 2017 [22,23]. The reason for the decrease in the number of *rep*-families in enterococcal isolates may be due to the presence of plasmids that do not belong to the 21 *rep*-families or due to unknown conditions that led to an instability and the subsequent loss of plasmids.

The predominant *rep*-family detected in the strains was *rep*_9_, represented by the pCF10 prototype, one of the pheromone-responsive plasmids in enterococci. As pheromone-responsive plasmids have been found almost exclusively in *E. faecalis* [10,24], the prevalence of this plasmid in this species was not unexpected. However, the predominance of *rep*_9_ in *E. faecium* isolates was surprising and could be due to the insufficient study of a wide variety of enterococci from poultry sources. The small sampling of *rep*-families from poultry *E. faecalis* and *E. faecium* previously reported *rep*_0_, *rep*_2_, and *rep*_9_ in *E. faecalis,* with a variety of *rep*-families (*rep*_2_, *rep*_3_, *rep*_4_, *rep*_5_, *rep*_6_, *rep*_7_, *rep*_14_, *rep*_17_) found in *E. faecium* [10,25]. Because of the nature of poultry production, such as the number of birds and environmental conditions pre-processing, it is not unreasonable that the opportunity for the transfer of plasmid *rep*-families is far greater in food animal production than clinical medicine, which may also account for the different *rep*-families in enterococci from poultry.

Both *E. faecalis* and *E. faecium* contained *rep*-families that were restricted to one or the other species in this study. *Rep*_7_ and *rep*_17_ were found exclusively in *E. faecalis,* while *rep*_5_ and *rep*_8_ were only found in *E. faecium*. Prototype pUSA02 (*rep*_7_) is characterized as a broad-host range plasmid that has been detected primarily in clinical *E. faecalis* [15,26,27], while prototype pRUM (*rep*_17_) is a conjugative, MDR plasmid originally isolated from a clinical *E. faecium* [28]. Surprisingly, none of the *E. faecium* in the present study were positive for *rep*_17_, which is a major deviation from results of some previous studies [10,29]. Both *rep*_5_ and *rep*_8_ were found only in *E. faecium* in this study. Both *rep*-families are described as having a narrow-host range, with *rep*_5_ predominating in *S. aureus,* while *rep*_8_ is another *rep*-family containing pheromone-responsive plasmids primarily found in *E. faecalis* [10]. *Rep*_5_ has previously been identified in *E. faecium* from chicken [25] and in a recent study of *S. aureus* from retail poultry from the U.S. [30], suggesting the genetic exchange of plasmid replicons among enterococci and staphylococci in poultry.

As most of the MDR isolates in this study included resistance to tetracycline, this antibiotic phenotype was used to determine the frequency of plasmid replicons associated with tetracycline resistance and was employed as a marker in conjugation studies. Interestingly, only for year 2005 was tetracycline resistance associated with the presence of a plasmid replicon. Although the specific *rep*-families associated with tetracycline resistance were not identified, 2005 was also the year with the highest number of different *rep*-families detected. The transfer of tetracycline resistance and *rep*_17_ using conjugation indicated a possible linkage between the antibiotic and *rep* type in a previous study [10]. It is also possible that some or more plasmid replicons for that year harbored tetracycline resistance genes. A definitive reason for the association of tetracycline resistance with the *rep*-family in this study was not determined.

Characterizing the mobility of plasmids is fundamental to understanding the epidemiology of plasmid-encoded antimicrobial resistance. In the present study, conjugation was performed to see if plasmids present in enterococcal isolates were transmissible which would enable determination of the potential transmissibility of the traits encoded on the enterococcal plasmids from animal sources to human sources. The transfer of plasmid replicons using conjugation, which was conducted using an *E. faecalis* recipient, was seen in the *E. faecalis* donors but not in *E. faecium,* which is indicative of the narrow-host range of some of the *rep*-families, and that intraspecies transfer is preferred over interspecies transfer [31]. Moreover, not all plasmids of the *rep*-families tested were transferred, which agrees with the previous analysis that revealed how some of the plasmids are non-transmissible [32]. Eight *rep*-families were successfully transferred from *E. faecalis* donors to the *E. faecalis* recipient, and some of these *rep*-families included *rep*_9_-containing pheromone-responsive plasmids such as pAD1 and pPD1—in addition to pCF10 discussed above—and the unique *rep*, which contains pMG1, a pheromone-independent conjugative plasmid. Pheromone-responsive plasmids, well-described in *E. faecalis*, transfer at a high frequency using aggregation substances for the clumping of donor and recipient cells; non-pheromone-responding plasmids are also known to transfer efficiently between different *Enterococcus* species [24,33]. There were four *rep*-families that were not transferred from *E. faecalis* donors to the *E. faecalis* recipient, and these included *rep*_1_, which contains pIP501, pAMβ1, and pSM19035, as well as *rep*_2_, which contains pRE25. These broad-host range conjugative plasmids do not transfer well in broth suspensions and transfer at a lower frequency on solid surfaces as compared to pheromone-responding plasmids and pMG1-related, pheromone-independent conjugative plasmids that transfer well on solid and in liquid, as reflected in our mating results [24,33].

## 5. Conclusions

This study clearly demonstrated that MDR *E. faecalis* and *E. faecium* from poultry contain multiple and diverse replicons. A shared pool of *rep*-families was evident, but a portion of *rep*-families was also unique to one or the other species. Although further testing with an *E. faecium* recipient is needed to test the host range, some of the replicons transferred to *E. faecalis* provide a mechanism for the spread of antimicrobial resistance and other genes among the enterococci. As the poultry carcass rinsates used in this study were from poultry processing facilities that provide poultry for human consumption, further studies on the prevalence, distribution, and transfer of the *rep*-families of enterococci from poultry and on the impact that these plasmid replicons have on human health are warranted.

## Figures and Tables

**Table 1:** Presence and prevalence of replicon families and plasmid prototypes among *Enterococcus faecalis* and *E. faecium* from 2004–2011.

		No. Isolates (%)	
Rep Family	Plasmid Prototype	*E. faecalis* (*n* = 47)	*E. faecium* (*n* = 110)
1	pIP501	1 (2.1)	7 (6.4)
2	pRE25	2 (4.3)	9 (8.2)
3	pAW63	19 (40.4)	26 (23.6)
4	pMBB1	ND	ND
5	pSAS/pN315	ND	5 (4.5)
6	pS86	9 (19.1)	12 (10.9)
7	pUSA02	1 (2.1)	ND
8	pAM373	ND	3 (2.7)
9	pCF10	32 (68.1)	44 (40)
10	pIM13	ND	ND
10b *	pSK6	ND	ND
11	pEF1071	13 (27.7)	26 (23.6)
12 *	pBMB67	ND	ND
13	pC194	ND	ND
14	pRI	5 (10.6)	9 (8.2)
15	pUSA03	ND	ND
16	pSAS	ND	ND
17	pRUM	1 (2.1)	ND
18	pEF418	3 (6.4)	7 (6.4)
19	pUB101	9 (19.1)	13 (11.8)
Unique	pMG1(pHTβ)	3 (6.4)	3 (2.7)

* PCR performed without positive control. ND = none detected.

**Table 2:** Distribution of plasmid replicons and families among multidrug resistant *Enterococcus faecalis* and *Enterococcus faecium* from 2004.

Species	Resistance Profile ^a^	No. Resistances	Plasmid Replicon	Replicon Family
*Enterococcus faecalis* (*n* = 11)	Bac Lin Nit	3	pCF10	9
	Bac Lin Tet Tyl	4	pCF10	9
	Bac Ery Lin Tyl	4	pCF10	9
	Gen Kan Lin Tet	4	pCF10	9
	Gen Kan Lin Tet	4	pCF10	9
	Kan Lin Str Tet	4	pCF10	9
	Bac Ery Lin Nit Tet Tyl	6	pRE25, pAW63, pEF1071	2, 3, 11
	Bac Flv Gen Kan Lin Tet	6	pAW63	3
	Bac Gen Kan Lin Str Tet	6	pAW63	3
	Ery Gen Kan Lin Tet Tyl	6	pCF10	9
	Bac Ery Kan Lin Str Tet Tyl	7	pAW63, pCF10	3, 9
*Enterococcus faecium* (*n* = 31)	Bac Cip Flv	3	pAW63, pCF10	3, 9
	Bac Flv Str	3	pCF10	9
	Bac Flv Tet	3	pEF1071	11
	Bac Lin Nit	3	pAW63, pCF10, pEF1071	3, 9, 11
	Cip Lin Nit	3	pAM373	8
	Lin Nit Pen	3	pAW63, pCF10, pUB101	3, 9, 19
	Lin Nit Tet	3	pRE25, pAW63, pMG1(pHTβ)	2, 3, Unique
	Lin Nit Tet	3	pAW63	3
	Str Syn Tet	3	pUB101	19
	Bac Cip Lin Nit	4	pEF1071	11
	Bac Flv Lin Syn	4	pAW63, pCF10	3,9
	Bac Lin Nit Pen	4	pSAS	5
	Cip Ery Flv Syn	4	pAW63, pCF10	3, 9
	Cip Lin Nit Pen	4	pAW63, pAM373, pCF10, pEF1071	3, 8, 9, 11
	Cip Lin Str Tet	4	pAW63, pCF10	3, 9
	Cip Lin Pen Tet	4	pCF10, pEF1071	9, 11
	Lin Str Syn Tet	4	pAW63, pUB101	3, 19
	Bac Cip Flv Lin Tet	5	pAW63, pSAS, pCF10	3, 5, 9
	Cip Lin Nit Syn Tet	5	pCF10	9
	Cip Lin Nit Syn Tet	5	pRI	14
	Bac Ery Flv Kan Lin Tet	6	pSAS	5
	Bac Flv Lin Str Syn Tet	6	pSAS, pCF10	5, 9
	Bac Lin Nit Pen Syn Tet	6	pAW63, pEF1071	3, 11
	Bac Lin Nit Pen Syn Tet	6	pAW63	3
	Bac Cip Flv Lin Nit Syn Tet	7	pCF10	9
	Bac Cip Lin Nit Str Syn Tet	7	pRE25, pCF10	2, 9
	Bac Flv Lin Nit Pen Syn Tet	7	pEF1071	11
	Ery Gen Kan Lin Nit Pen Tyl	7	pCF10	9
	Bac Cip Flv Gen Kan Lin Nit Pen	8	pRE25, pAW63, pSAS, pRI	2, 3, 5, 14
	Bac Cip Flv Lin Nit Pen Syn Tet	8	pAW63,	3
	Bac Dap Gen Kan Lin Nit Pen Syn	8	pCF10	9

^a^ Bac = bacitracin, Cip = ciprofloxacin, Dap = daptomycin, Ery = erythromycin, Flv = flavomycin, Gen = gentamicin, Kan = kanamycin, Lin = lincomycin, Nit = nitrofurantoin, Pen = penicillin, Str = streptomycin, Syn = Synercid (Quinupristin/Dalfopristin), Tet = tetracycline, Tyl = tylosin.

**Table 3:** Distribution of plasmid replicons and families among multidrug resistant *Enterococcus faecalis* and *Enterococcus faecium* from 2005.

Species	Resistance Profile ^a^	No. Resistances	Plasmid Replicon	Replicon Family
*Enterococcus faecalis* (*n* = 29)	Bac Lin Tet	3	pMG1(pHTβ)	Unique
	Cip Lin Str	3	pCF10	3, 6, 9
	Gen Kan Lin	3	pCF10	9
	Gen Kan Tet	3	pAW63, pS86, pCF10, pEF1071, pEF418, pUB101	3, 6, 9, 11, 18, 19
	Gen Kan Tet	3	pAW63, pS86, pEF1071, pUB101	3, 6, 11, 19
	Lin Str Tet	3	pEF1071, pRI	11, 14
	Bac Gen Kan Tet	4	pAW63, pCF10	3, 9
	Bac Gen Kan Tet	4	pCF10	9
	Bac Gen Kan Tet	4	pRI	14
	Bac Gen Kan Tet	4	pRI	14
	Bac Lin Str Tet	4	pAW63, pS86, pCF10, pMG1(pHTβ), pRUM, pUB101	3, 6, 9, Unique, 17, 19
	Ery Kan Lin Tet	4	pAW63, pS86, pCF10, pUB101	3, 6, 9, 19
	Ery Kan Lin Tyl	4	pMG1(pHTβ)	Unique
	Ery Lin Tet Tyl	4	pS86, pEF1071	6, 11
	Gen Kan Lin Tet	4	pCF10	9
	Gen Kan Lin Tet	4	pCF10	9
	Kan Lin Tet Tyl	4	pCF10, pRI	9, 14
	Bac Gen Kan Lin Nit	5	pAW63, pCF10	3, 9
	Bac Gen Kan Lin Tet	5	pAW63, pS86, pEF1071	3, 9, 11
	Bac Ery Lin Tet Tyl	5	pAW63, pCF10, pEF1071	3, 9, 11
	Bac Ery Lin Tet Tyl	5	pCF10	9
	Ery Lin Str Tet Tyl	5	pAW63, pUSA02, pCF10, pEF1071, pUB101	3, 7, 9, 11, 19
	Ery Lin Nit Tet Tyl	5	pAW63	3
	Bac Gen Kan Lin Str Tet	6	pRI, pEF418	14, 18
	Ery Gen Kan Lin Tet Tyl	6	pAW63, pS86, pCF10, pEF1071, pUB101	3, 6, 9, 11, 19
	Bac Ery Gen Kan Lin Tet Tyl	7	pAW63, pS86, pCF10, pEF1071, pUB101	3, 6, 9, 11, 19
	Bac Ery Gen Kan Lin Tet Tyl	7	pAW63, pCF10	3,9
	Bac Ery Gen Kan Lin Tet Tyl	7	pCF10	9
	Bac Ery Gen Kan Lin Str Tet Tyl	8	pCF10, pRI	9, 14
*Enterococcus faecium* (*n* = 25)	Bac Flv Str	3	pCF10, pEF1071	9, 11
	Lin Nit Pen	3	pCF10	9
	Lin Nit Pen	3	pEF1071	11
	Lin Nit Tet	3	pCF10, pUB101	9, 19
	Lin Pen Tet	3	pRI	14
	Bac Cip Lin Nit	4	pAW63, pCF10, pEF418	3, 9, 18
	Bac Ery Lin Tet	4	pAW63	3
	Bac Flv Lin Tet	4	pCF10	9
	Cip Lin Nit Tet	4	pAW63, pCF10, pEF1071	3, 9, 11
	Gen Kan Lin Pen	4	pAW63, pRI	3, 14
	Lin Pen Syn Tet	4	pRI	14
	Lin Str Syn Tet	4	pAW63, pCF10, pUB101	3, 9, 19
	Dap Lin Nit Syn Tet	5	pAW63, pS86, pCF10, pEF1071, pUB101	3, 6, 9, 11, 19
	Bac Flv Lin Pen Tet	5	pCF10, pEF1071	9, 11
	Bac Cip Lin Nit Pen Tet	6	pAW63, pS86, pMG1(pHTβ)	3, 6, Unique
	Bac Cip Lin Pen Syn Tet	6	pMG1(pHTβ)	Unique
	Bac Lin Nit Pen Syn Tet	6	pAW63, pCF10	3, 9
	Ery Lin Nit Syn Tet Tyl	6	pRI	14
	Gen Kan Lin Nit Pen Tet	6	pAW63, pS86, pEF1071	3, 6, 11
	Bac Cip Lin Nit Pen Syn Tet	7	pAW63, pEF1071	3, 11
	Cip Gen Kan Lin Nit Pen Syn	7	pCF10	9
	Ery Lin Nit Pen Syn Tet Tyl	7	pS86	6
	Bac Ery Flv Kan Lin Pen Str Tyl	8	pAW63	3
	Gen Kan Lin Nit Pen Str Syn Tet	8	pS86	6
	Bac Chl Cip Dap Flv Gen Kan Nit Pen Str Tet Tyl	12	pRI	14

^a^ Bac = bacitracin, Chl = chloramphenicol, Cip = ciprofloxacin, Dap = daptomycin, Ery = erythromycin, Flv = flavomycin, Gen = gentamicin, Kan = kanamycin, Lin = lincomycin, Nit = nitrofurantoin, Pen = penicillin, Str = streptomycin, Syn = Synercid (Quinupristin/Dalfopristin), Tet = tetracycline, Tyl = tylosin.

**Table 4:** Distribution of plasmid replicons and families among multidrug resistant *Enterococcus faecalis* and *Enterococcus faecium* from 2006–2007.

Year	Species	Resistance Profile ^a^	No. Resistances	Plasmid Replicon	Replicon Family
2006	*Enterococcus faecalis* (*n* = 3)	Gen Kan Tet	3	pEF1071, pUB101	11, 19
		Lin Nit Tet	3	pIP501	1
		Ery Lin Tet Tyl	4	pCF10	9
	*Enterococcus faecium* (*n* = 19)	Flv Lin Syn	3	pCF10, pUB101	9, 19
		Lin Nit Tet	3	pIP501, pRE25	1, 2
		Lin Pen Tet	3	pRE25	2
		Lin Syn Tet	3	pS86, pEF1071, pEF418, pUB101	6, 11, 18, 19
		Ery Pen Str Tet	4	pCF10	9
		Lin Nit Syn Tet	4	pRE25	2
		Lin Nit Syn Tet	4	pCF10, pEF418	9, 18
		Cip Nit Pen Syn Tet	5	pEF1071	11
		Gen Kan Lin Nit Tet	5	pCF10	9
		Lin Nit Pen Syn Tet	5	pCF10	9
		Ery Flv Lin Syn Tet Tyl	6	pS86	6
		Ery Gen Lin Nit Tet Tyl	6	pAW63, pEF1071	3, 11
		Cip Ery Gen Lin Syn Tet Tyl	7	pIP501	1
		Cip Ery Lin Nit Syn Tet Tyl	7	pCF10	9
		Cip Gen Kan Lin Nit Syn Tet	7	pIP501	1
		Cip Ery Lin Pen Syn Tet Tyl	7	pIP501	1
		Ery Flv Lin Nit Syn Tet Tyl	7	pCF10	9
		Ery Gen Kan Lin Str Syn Tet Tyl	8	pRE25, pS86, pEF1071	2, 6, 11
		Ery Kan Lin Nit Str Syn Tet Tyl	8	pS86, pEF1071, pUB101	6, 11, 19
2007	*Enterococcus faecalis* (*n* = 3)	Cip Flv Lin	3	pRE25, pS86, pEF1071	2, 6, 11
		Ery Lin Tyl	3	pCF10	9
		Flv Lin Tet	3	pEF418	18
	*Enterococcus faecium* (*n* = 14)	Lin Nit Pen	3	pEF418	18
		Lin Syn Tet	3	pUB101	19
		Cip Flv Lin Tet	4	pS86	6
		Cip Lin Nit Tet	4	pUB101	19
		Flv Lin Syn Tet	4	pCF10	9
		Lin Nit Pen Tet	4	pCF10, pEF1071	9, 11
		Cip Ery Lin Nit Tyl	5	pAM373	8
		Cip Lin Pen Syn Tet	5	pEF418	18
		Gen Kan Lin Nit Tet	5	pCF10	9
		Cip Flv Lin Nit Syn Tet	6	pRE25	2
		Ery Gen Kan Lin Tet Tyl	6	pEF418	18
		Ery Lin Pen Syn Tet Tyl	6	pEF418	18
		Flv Gen Kan Lin Nit Tgc	6	pRE25	2
		Cip Flv Lin Nit Pen Str Syn Tet Tgc	9	pS86	6

^a^ Cip = ciprofloxacin, Ery = erythromycin, Flv = flavomycin, Gen = gentamicin, Kan = kanamycin, Lin = lincomycin, Nit = nitrofurantoin, Pen = penicillin, Str = streptomycin, Syn = Synercid (Quinupristin/Dalfopristin), Tet = tetracycline, Tgc = tigecycline, Tyl = tylosin.

**Table 5:** Distribution of plasmid replicons and families among multidrug resistant *Enterococcus faecalis* and *Enterococcus faecium* from 2008–2011.

Year	Species	Resistance Profile ^a^	No. Resistances	Plasmid Replicon	Replicon Family
2008	*Enterococcus faecalis* (*n* = 1)	Gen Kan Lin Tet	4	pUB101	19
	*Enterococcus faecium* (*n* = 6)	Cip Flv Lin Tet	4	pCF10	9
		Cip Lin Nit Syn Tet	5	pIP501	1
		Dap Flv Lin Nit Tet	5	pCF10	9
		Lin Nit Pen Syn Tet	5	pIP501	1
		Cip Flv Lin Nit Pen Syn Tet	7	pEF1071	11
		Cip Ery Flv Lin Nit Pen Syn Tet Tyl	9	pIP501	1
2009	*Enterococcus faecium* (*n* = 1)	Lin Nit Syn Tet	4	pCF10	9
2010	*Enterococcus faecium* (*n* = 5)	Cip Lin Nit	3	pEF1071, pRI	11, 14
		Cip Lin Nit Pen	4	pEF1071	11
		Lin Str Syn Tet	4	pEF1071	11
		Cip Lin Nit Pen Syn Tet	6	pEF1071	11
		Ery Lin Nit Pen Str Syn Tet Tyl	8	pRI	14
2011	*Enterococcus faecium* (*n* = 9)	Lin Syn Tet	3	pCF10, pUB101	9, 19
		Ery Lin Syn Tet	4	pEF1071	11
		Ery Lin Syn Tet	4	pCF10	9
		Lin Str Syn Tet	4	pS86, pCF10, pUB101	6, 9, 19
		Lin Nit Pen Syn Tet	5	pCF10	9
		Cip Lin Nit Pen Syn Tet	6	pCF10	9
		Cip Lin Nit Pen Str Syn Tet	7	pCF10	9
		Ery Gen Kan Lin Syn Tet Tyl	7	pEF1071	11
		Cip Ery Kan Lin Nit Str Tet Tyl	8	pCF10	9

^a^ Cip = ciprofloxacin, Dap = daptomycin, Ery = erythromycin, Flv = flavomycin, Gen = gentamicin, Kan = kanamycin, Lin = lincomycin, Nit = nitrofurantoin, Pen = penicillin, Str = streptomycin, Syn = Synercid (Quinupristin/Dalfopristin), Tet = tetracycline, Tyl = tylosin.

**Table 6:** Transfer of plasmid replicons and replicon families.

Donor ID	Species	Plasmid Replicon Profile	Replicon Family Profile	Transconjugant Replicon Profile	Replicon Family Transferred
ARS820	*E. faecalis*	pAW63	3	pAW63	3
ARS323	*E. faecalis*	pUB101	19	pUB101	19
ARS808	*E. faecalis*	pAW63, pS86, pCF10, pMG1(pHTβ), pRUM, pUB101	3, 6, 9, Unique, 17, 19	pAW63, pS86, pCF10, pRUM, pUB101	3, 6, 9, 17, 19
ARS828	*E. faecalis*	pAW63, pUSA02, pCF10, pEF1071, pUB101	3, 7, 9, 11, 19	pAW63, pUSA02, pUB101	3, 7, 19
ARS515	*E. faecalis*	pRE25, pAW63, pEF1071	2, 3, 9	pAW63	3
ARS796	*E. faecalis*	pRI	14	Colonies, no replicon transfer	N/A
ARS858	*E. faecalis*	pMG1(pHTβ)	Unique	pMG1(pHTβ)	Unique
ARS795	*E. faecalis*	pAW63, pS86, pCF10, pEF1071, pEF418, pUB101	3, 6, 9, 11, 18, 19	pS86	6
ARS151	*E. faecalis*	pEF418	18	pEF418	18
ARS085	*E. faecalis*	pIP501	1	Colonies, no replicon transfer	N/A

N/A = not applicable, no colonies were detected on plates. No transfer was detected for pRE25, pEF1071, pRI, pSAS, pAM373, and pIP501.

## Data Availability

Not applicable.

## Supplementary Materials

The following supporting information can be downloaded at:https://www.mdpi.com/article/10.3390/microorganisms10061244/s1, Table S1: Multidrug resistance patterns of *Enterococcus faecalis* and *Enterococcus faecium* from poultry.
