# Supplementary material for: Distribution and Transfer of Plasmid Replicon Families among Multidrug-Resistant Enterococcus faecalis and Enterococcus faecium from Poultry
Source: Microorganisms. 2022 Jun 17;10(6):1244. doi: 10.3390/microorganisms10061244 (PMC9228330; doi:10.3390/microorganisms10061244)
Supplement: Supplementary file 1 [file microorganisms-10-01244-s001.zip › microorganisms-1766479-supplementary.pdf]

Supplementary Table S1. Multidrug resistance patterns of *Enterococcus faecalis* and *Enterococcus faecium* from poultry.

	No. isolates (%)	
Resistance pattern[a, b]	*E. faecalis* (n=47)	*E. faecium* (n=110)
Bac Cip Flv		1 (0.9)
Bac Flv Str		2 (1.8)
Bac Flv Tet		1 (0.9)
Bac Lin Nit	1 (2.1)	1 (0.9)
Bac Lin Tet	1 (2.1)	
Cip Lin Nit		1 (0.9)
Cip Flv Lin	1 (2.1)	
Cip Lin Nit		1 (0.9)
Cip Lin Str	1 (2.1)	
Ery Lin Tyl	1 (2.1)	
Flv Lin Syn		1 (0.9)
Flv Lin Tet	1 (2.1)	
Gen Kan Lin	1 (2.1)	
Gen Kan Tet	3 (6.4)	
Lin Nit Pen		4 (3.6)
Lin Nit Tet	1 (2.1)	4 (3.6)
Lin Pen Tet		2 (1.8)
Lin Str Tet	1 (2.1)	
Lin Syn Tet		3 (2.7)
Str Syn Tet		1 (0.9)
Bac Cip Lin Nit		2 (1.8)
Bac Ery Lin Tet		1 (0.9)
Bac Ery Lin Tyl	1 (2.1)	
Bac Flv Lin Syn		1 (0.9)
Bac Flv Lin Tet		1 (0.9)
Bac Gen Kan Tet	4 (8.5)	
Bac Lin Nit Pen		1 (0.9)
Bac Lin Str Tet	1 (2.1)	
Bac Lin Tet Tyl	1 (2.1)	
Cip Lin Nit Pen		1 (0.9)
Cip Lin Nit Tet		1 (0.9)
Cip Lin Pen Tet		1 (0.9)
Cip Lin Str Tet		1 (0.9)
Cip Ery Flv Syn		1 (0.9)
Cip Ery Lin Nit Tyl		1 (0.9)
Cip Flv Lin Tet		2 (1.8)
Cip Lin Nit Pen		1 (0.9)
Cip Lin Nit Tet		1 (0.9)
Ery Kan Lin Tet	1 (2.1)	
Ery Kan Lin Tyl	1 (2.1)	

Ery Lin Tet Tyl	1 (2.1)	
Ery Lin Syn Tet		2 (1.8)
Ery Lin Tet Tyl	1 (2.1)	
Ery Pen Str Tet		1 (0.9)
Flv Lin Syn Tet		1 (0.9)
Gen Kan Lin Pen		1 (0.9)
Gen Kan Lin Tet	5 (10.6)	
Gen Kan Lin Nit Tet		2 (1.8)
Kan Lin Str Tet	1 (2.1)	
Kan Lin Tet Tyl	1 (2.1)	
Lin Nit Pen Tet		4 (3.6)
Lin Pen Syn Tet		1 (0.9)
Lin Str Syn Tet		4 (3.6)
Bac Cip Flv Lin Tet		1 (0.9)
Bac Ery Lin Tet Tyl	2 (4.3)	
Bac Flv Lin Pen Tet		1 (0.9)
Bac Gen Kan Lin Nit	1 (2.1)	
Bac Gen Kan Lin Tet	1 (2.1)	
Cip Lin Nit Syn Tet		3 (2.7)
Cip Lin Pen Syn Tet		1 (0.9)
Cip Nit Pen Syn Tet		1 (0.9)
Dap Lin Nit Syn Tet		1 (0.9)
Dap Flv Lin Nit Tet		1 (0.9)
Ery Lin Nit Tet Tyl	1 (2.1)	
Ery Lin Str Tet Tyl	1 (2.1)	
Lin Nit Pen Syn Tet		3 (2.7)
Bac Cip Lin Nit Pen Tet		1 (0.9)
Bac Cip Lin Pen Syn Tet		1 (0.9)
Bac Ery Flv Kan Lin Tet		1 (0.9)
Bac Ery Lin Nit Tet Tyl	1 (2.1)	
Bac Flv Gen Kan Lin Tet	1 (2.1)	
Bac Flv Lin Str Syn Tet		1 (0.9)
Bac Gen Kan Lin Str Tet	2 (4.3)	
Bac Lin Nit Pen Syn Tet		3 (2.7)
Cip Flv Lin Nit Syn Tet		1 (0.9)
Cip Lin Nit Pen Syn Tet		2 (1.8)
Ery Gen Kan Lin Tet Tyl	1 (2.1)	
Ery Lin Nit Syn Tet Tyl		1 (0.9)
Ery Flv Lin Syn Tet Tyl		1 (0.9)
Ery Gen Kan Lin Tet Tyl	1 (2.1)	1 (0.9)
Ery Gen Lin Nit Tet Tyl		1 (0.9)
Ery Lin Pen Syn Tet Tyl		1 (0.9)
Gen Kan Lin Nit Pen Tet		1 (0.9)
Flv Gen Kan Lin Nit Tgc		1 (0.9)

Bac Cip Flv Lin Nit Syn Tet		1 (0.9)
Bac Cip Lin Nit Pen Syn Tet		1 (0.9)
Bac Cip Lin Nit Str Syn Tet		1 (0.9)
Bac Ery Gen Kan Lin Tet Tyl	3 (6.4)	
Bac Ery Kan Lin Str Tet Tyl	1 (2.1)	
Bac Flv Lin Nit Pen Syn Tet		1 (0.9)
Cip Gen Kan Lin Nit Pen Syn		1 (0.9)
Cip Gen Kan Lin Nit Syn Tet		1 (0.9)
Cip Ery Lin Nit Syn Tet Tyl		1 (0.9)
Cip Ery Gen Lin Syn Tet Tyl		1 (0.9)
Cip Ery Lin Pen Syn Tet Tyl		1 (0.9)
Cip Flv Lin Nit Pen Syn Tet		1 (0.9)
Cip Lin Nit Pen Str Syn Tet		1 (0.9)
Ery Gen Kan Lin Nit Pen Tyl		1 (0.9)
Ery Lin Nit Pen Syn Tet Tyl		1 (0.9)
Ery Flv Lin Nit Syn Tet Tyl		1 (0.9)
Ery Gen Kan Lin Syn Tet Tyl		1 (0.9)
Bac Cip Flv Gen Kan Lin Nit Pen		1 (0.9)
Bac Cip Flv Lin Nit Pen Syn Tet		1 (0.9)
Bac Dap Gen Kan Lin Nit Pen Syn		1 (0.9)
Bac Ery Flv Kan Lin Pen Str Tyl		1 (0.9)
Bac Ery Gen Kan Lin Str Tet Tyl	1 (2.1)	
Cip Ery Kan Lin Nit Str Tet Tyl		1 (0.9)
Ery Gen Kan Lin Str Syn Tet Tyl		1 (0.9)
Ery Kan Lin Nit Str Syn Tet Tyl		1 (0.9)
Ery Lin Nit Pen Str Syn Tet Tyl		1 (0.9)
Gen Kan Lin Nit Pen Str Syn Tet		1 (0.9)
Cip Ery Flv Lin Nit Pen Syn Tet Tyl		1 (0.9)
Cip Flv Lin Nit Pen Str Syn Tet Tgc		1 (0.9)
Bac Chl Cip Dap Flv Gen Kan Nit Pen Str Tet Tyl		1 (0.9)

[a]Bac=bacitracin, Chl=chloramphenicol, Cip=ciprofloxacin, Dap=daptomycin, Ery=erythromycin, Flv=flavomycin, Gen=gentamicin, Kan=kanamycin, Lin=lincomycin Nit=nitrofurantoin, Pen=penicillin, Str=streptomycin, Syn=Synercid (Quinupristin/Dalfopristin), Tet=tetracycline, Tyl=tylosin

[b]Font colors: Black=*E. faecalis*, Red=*E. faecium*, Blue=*E. faecalis* and *E. faecium*
